# Evaluation of Circulating Cardiovascular Biomarker Levels for Early Detection of Congenital Heart Disease in Newborns in Sweden

**DOI:** 10.1001/jamanetworkopen.2020.27561

**Published:** 2020-12-02

**Authors:** Henning Clausen, Elisabeth Norén, Salla Valtonen, Aki Koivu, Mikko Sairanen, Petru Liuba

**Affiliations:** 1 Regional Paediatric Cardiology Service, Department of Paediatrics, University Teaching Hospital Ryhov, Jönköping, Sweden; 2 Children’s Heart Centre, Scania University Hospital and Lund University, Lund, Sweden; 3 Clinical Laboratory Science, University Teaching Hospital Ryhov, Jönköping, Sweden; 4 Department of Chemistry, University of Turku, Turku, Finland; 5 Research and Development Division, PerkinElmer, Turku, Finland

## Abstract

**Importance:**

Congenital heart disease (CHD) is the most common congenital malformation in humans worldwide. Circulating cardiovascular biomarkers could potentially improve the early detection of CHD, even in asymptomatic newborns.

**Objectives:**

To assess the performance of a dried blood spot (DBS) test to measure the cardiovascular biomarker amino terminal fragment of the prohormone brain-type natriuretic peptide (NT-proBNP) levels in newborns and to compare DBS with standard EDTA analysis in control newborns during the first week of life.

**Design, Setting, and Participants:**

This diagnostic study was conducted in a single regional pediatric service in southern Sweden. Healthy, term neonates born between July 1, 2018, and May 31, 2019, were prospectively enrolled and compared against retrospectively identified newborns with CHD born between September 1, 2003, and September 30, 2019. Neonates who required inpatient treatment beyond the standard postnatal care were excluded.

**Exposure:**

New DBS test for NT-proBNP quantification in newborns that used 3 μL of blood vs the current screening standard.

**Main Outcomes and Measures:**

Performance of the new test and when combined with pulse oximetry screening was measured by receiver operating characteristic curve analysis. Performance of the new test and EDTA screening was compared using Pearson linear correlation analysis.

**Results:**

The DBS samples of 115 neonates (81 control newborns and 34 newborns with CHD, of whom 63 were boys [55%] and the mean [SD] gestational age was 39.6 [1.4] weeks) were analyzed. The new NT-proBNP test alone identified 71% (n = 24 of 34) of all CHD cases and 68% (n = 13 of 19) of critical CHD cases as soon as 2 days after birth. Detection of any CHD type improved to 82% (n = 28 of 34 newborns) and detection of critical CHD improved to 89% (n = 17 of 19 newborns) when combined pulse oximetry screening and NT-proBNP test results were used. Performance of the NT-proBNP test was excellent when control newborns were matched to newborns with CHD born between July 1, 2018, and May 31, 2019 (area under the curve, 0.96; SE, 0.027; 95% CI, 0.908-1.0; asymptotic *P* < .05).

**Conclusions and Relevance:**

This study found that NT-proBNP assay using minimal DBS samples appears to be timely and accurate in detecting CHD in newborns and to discriminate well between healthy newborns and newborns with various types of CHD. This finding warrants further studies in larger cohorts and highlights the potential of NT-proBNP to improve neonatal CHD screening.

## Key Points

**Question:**

Could a new test that measures the cardiovascular biomarker amino terminal fragment of the prohormone brain-type natriuretic peptide (NT-proBNP) using dried blood samples be used to identify neonates born with congenital heart disease (CHD)?

**Findings:**

In this diagnostic study of 115 newborns in Sweden, a fully automated diagnostic assay for NT-proBNP quantification was developed that used 3 μL of dried blood spot samples. This new test discriminated well between newborns with CHD and control newborns.

**Meaning:**

Results of this study suggest that the new NT-proBNP test warrants further evaluation in larger cohorts to assess its ability for universal detection of CHD through established newborn screening programs.

## Teaser

This diagnostic study assesses the use of dried blood samples to detect prohormone brain-type natriuretic peptide for diagnosis of congenital heart disease in neonates in Sweden.

## Introduction

Congenital heart disease (CHD) affects approximately 1 in 125 newborns and is the most common congenital malformation in humans, causing substantial morbidity and mortality rates worldwide.^1,2,3^ This prevalence has led to the development of highly specialized services with favorable surgical mortality outcomes, which are largely attributed to centralized pediatric cardiac centers and dedicated intensive care units.^4^

To improve the early identification of CHD, maternity services have implemented prenatal ultrasonography screening programs. Prenatal CHD detection has been associated with improved perinatal care in critical cases, but these screening efforts remain imperfect even in high-income countries.^5,6,7,8,9,10^ Delayed CHD diagnosis is associated with increased perioperative morbidity and mortality.^11,12^ Critical CHD remains associated with risk of sudden cardiovascular collapse after discharge from maternity services.^11,13^ Early postnatal detection of CHD can be improved through standardized clinical examination protocols, but evaluation of newborns with CHD requires clinical experience because of neonates’ transition from fetal to postnatal circulation.^14^ Aortic arch obstructions from a slowly closing ductus arteriosus without apparent femoral pulse deficit or only mild cyanosis may go unnoticed.^15^ Pulse oximetry (POX) screening has been recommended as a complement to clinical examination because it may help to detect critical CHD.^16,17,18^ Pulse oximetry screening is based on measuring oxygen saturations in the right arm (preductal) and lower extremities (postductal) approximately 6 to 24 hours after birth to identify cyanosis (<95% saturation) or a greater than 3% saturation difference between preductal and postductal measurements. If the newborn has a positive screen result (in this study, *positive* is defined as a problem identified, whereas *negative* indicates no problem), prompt neonatal assessment and subsequent echocardiographic evaluation are paramount. In this setting, some neonates with even minor hypoxemia owing to delays in adaptation from the fetal circulation or those with primary pulmonary disease will inevitably have positive screen results. This finding has raised questions regarding the ability of POX to detect critical CHD.^19^ No prospective studies have been performed on the POX screening method used solely for identifying pulmonary disease.^20,21^ Although concerns remain that POX screening is imperfect for uncovering CHD lesions involving a duct-dependent systemic circulation, the method has become a widespread tool for detecting critical CHD in many health care systems.^15,22^

Given that screening for all types of CHD remains challenging, we focused on the established dried blood spot (DBS) method in newborns. The DBS analysis has been part of neonatal screening programs around the world for many decades and is mainly used to find inborn errors of metabolism.^23,24,25,26,27,28^

Circulating biomarkers of heart disease have been studied in adults with congestive heart disease and in small groups of infants and children with various cardiovascular pathologies, and natriuretic peptides have been implicated in the pathophysiological origins of heart failure. The amino terminal fragment of the prohormone brain-type natriuretic peptide (NT-proBNP) test has been used in adults with cardiac failure.^29^ Evidence has found that the NT-proBNP test is useful in infants who require neonatal intensive care, children with pulmonary hypertension and cardiomyopathies, and children with various types of CHD.^30,31,32,33,34,35,36,37^ Small studies on the use of natriuretic peptides in children have been published.^38,39,40^

To our knowledge, no study has addressed the NT-proBNP test as a universal tool for detecting CHD in newborns using DBS samples. This pilot study was conducted to validate a new DBS NT-proBNP assay. We aimed to compare DBS with standard EDTA analysis in healthy newborns (controls) during the first week of life. The DBS method was then used to test the hypothesis that CHD cases express substantially higher levels of NT-proBNP. We were particularly interested in neonates who were born with critical CHD and in those who were discharged home after birth without symptoms suggestive of CHD.

## Methods

This diagnostic study was conducted in a single regional pediatric service in the designated health care region of Jönköping in southern Sweden in accordance with the ethical principles of the Declaration of Helsinki.^41^ It was approved by the regional ethics committee of Linköping. Written informed consent was obtained from parents or guardians. We followed the Standards for Reporting of Diagnostic Accuracy (STARD) reporting guideline. Data collection was planned prospectively, and the data collection process and eligibility criteria are summarized in the eAppendix in the Supplement.

### CHD Background Evaluation and Newborn Enrollment

Before the study commenced, we reviewed national incidence and outcomes data of CHD from the 2009 to 2017 published reports by the Swedish Registry of Congenital Heart Disease.^42^ To better understand current standard screening practices, we obtained internal departmental audit data summaries from the pediatric service for the 5.5-year period (2008-2013) after POX screening implementation.

Recruitment for the study was advertised, and enrollment was based on convenience sampling. Timing of DBS screening using standard protocols coincided with routine newborn blood screening more than 48 hours after birth during the first week of life. We prospectively enrolled healthy, term newborns born from July 1, 2018, to May 31, 2019, and we excluded neonates who required inpatient treatment beyond the standard postnatal care according to local guidelines given that these newborns were not candidates for timely postnatal hospital discharge. We retrospectively identified newborns with CHD born between September 1, 2003, and September 30, 2019. Newborns who were scheduled for routine outpatient clinical reviews during this time frame were approached when their families showed interest. We had no prior knowledge of which newborns with CHD were scheduled for clinical reviews.

The CHD diagnosis was confirmed by echocardiography. Control newborns were followed-up for a minimum of 1 year using electronic patient records covering routine checkups and additional medical consultations. The laboratory staff was blinded to clinical details.

### NT-proBNP Test

To compare the new test with current screening standards, we offered the NT-proBNP assay, using approximately 500 μL of blood for EDTA sampling, to all parents of newborns in the control group at the time of DBS screening. EDTA blood analyses were performed based on previously published methods using the Elecsys proBNP II assay (Roche Diagnostics).^43^ A safety cutoff value chosen for the detection of CHD in newborns in the control group using same-day analyses was 12 000 ng/L, based on published references.^33,39^ Patients who exceeded this cutoff level were examined with echocardiography performed by a pediatric cardiologist (H.C.) within 24 hours. Stored DBS samples for CHD cases were retrieved from the national newborn screening laboratory biobank in Sweden. These samples were stored at 4 °C with 30% humidity, whereas subsequent samples were stored at –20 °C.

A fully automated immunoassay for NT-proBNP measurement from DBS samples was developed. Microtitration strips 96-well format (Nunc; ThermoFisher Scientific) were coated with anti-NT-proBNP mouse monoclonal IgG antibody (HyTest Ltd). Monoclonal tracer antibody (HyTest Ltd) was labeled with europium chelate. Excess amount of chelate was incubated with antibody overnight, and Eu-labeled antibody preparation was purified using a chromatography system (Äkta Prime; GE Healthcare) with a separation column (Superdex 200 10/300 GL gel filtration column; GE Healthcare). The final product was stabilized with diethylenetriaminepentaacetic acid-bis (stearylamide) and was filtered with a 0.22-μm sterile syringe filter (Millex-GV; Millipore). To prepare the DBS calibrators, we mixed artificial serum (0.9% [weight/volume] NaCl solution supplemented with a 350-μM sucrose solution) into the washed red blood cells (Diaserve Laboratories). The hematocrit in this mix was checked with a hematology analyzer (Coulter Ac · T diff Hematology Analyzer; Beckman Coulter). Red blood cell preparation was spiked with the following NT-proBNP concentrations: 0, 300, 1000, 3000, 10 000, and 30 000 ng/L. The blood spots were prepared by final dilutions of 75 μl per spot onto filter paper (Whatman 903; Whatman). The filter paper sheets were dried overnight and then packaged and sealed in airtight foil bags with 2-g silica desiccant packets (MiniPax; Multisorb Technologies Inc) for storage at –20 °C until use.

From the DBS samples or calibrators, 3.2-mm disks were punched (using DELFIA DBS Puncher 1296-071; PerkinElmer) with 3 μl of blood into wells coated previously with anti-NT-proBNP antibody. The plate was assayed with a high throughput batch analyzer (GSP Instrument; PerkinElmer). An elution buffer volume of 150 μl per well was added to 75 ng per well tracer antibody in 5 μl and, when plated, was incubated for 3 hours. Next, DBS samples were removed, the plate was washed 4 times, the inducer solution was added (200 μl/well), and the signal was measured.

### Statistical Analysis

Anonymized clinical data were collected and entered into an electronic database for statistical analysis using SPSS, version 26 (IBM); TIBCO Spotfire, version 7.11.1 (TIBCO Software Inc); and R, version 3.5.1 (R Foundation for Statistical Computing). Diagnoses were based on *International Statistical Classification of Diseases and Related Health Problems, Tenth Revision* codes and verified against electronic patient and echocardiographic records.^4,44^ We anticipated that 5% of control newborns and at least 25% of newborns with CHD would have NT-proBNP levels greater than 12 000 ng/L. Enrollment was at a 1:3 case to control ratio. To achieve 80% power with α = .05, we calculated the number of control newborns (n = 85) and newborns with CHD (n = 28) for a total of 113. In addition, NT-proBNP test data were logarithmically transformed to achieve symmetrical distribution and to allow for direct comparison with previously published references. Results were expressed in percentages, means (SD), or medians (interquartile range [IQR]). Pearson linear correlation analysis was used to compare the performance between the NT-proBNP test and EDTA screening. Bland-Altman analysis was performed to analyze agreement between the 2 screening tests. Performance of the NT-proBNP test and when it was combined with POX screening was measured by receiver operating characteristic (ROC) curve analysis. A 2-sided *P* < .05 was considered to be statistically significant.

## Results

**Figure 1.**
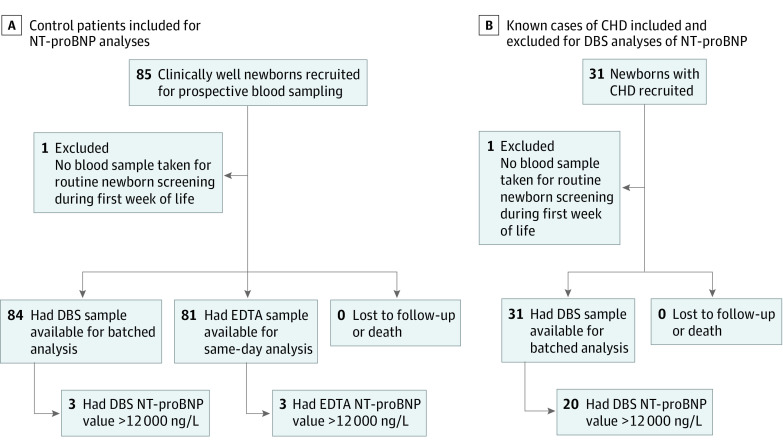
Study Flow Diagram for Control and Congenital Heart Disease (CHD) Groups A, Two of 3 newborns with dried blood spot (DBS) results greater than 12 000 ng/L had minor congenital heart disease (CHD) (ie, patent ductus arteriosus, ventricular septal defect), and these results were identical to those identified by EDTA blood analyses; 1 result was a false positive. Three asymptomatic newborns with minor CHD were identified by EDTA amino terminal fragment of the prohormone brain-type natriuretic peptide (NT-proBNP) analyses using the initial cutoff at 12 000 ng/L based on previous references. B, DBS sampling was accidentally delayed by several weeks after heart surgery in 1 case of transposition of the great arteries. Exclusion did not alter the overall results of the study.

The new DBS NT-proBNP assay was evaluated in 115 newborns, of which 81 were control and 34 were case patients, and 63 were boys (55%) and 52 were girls (45%) with a mean (SD) gestational age of 39.6 (1.4) weeks (Figure 1 and Table 1). Of the 34 patients with CHD, 3 newborns in the control group were found to have minor CHD. Neither gestational age nor Apgar scores at 1 to 10 minutes were substantially different between control newborns and those with CHD. The group with CHD compared with the control group had a higher percentage of male neonates (68% [n = 23 of 34] vs 49% [n = 40 of 81]; *P* = .11) and cesarean deliveries (21% [n = 7 of 34] vs 9% [n = 7 of 81]; *P* = .23), although this composition did not statistically significantly affect NT-proBNP test results. No neonates were lost to follow-up, and all were alive at the end of the study period (July 1, 2020); no additional cases of CHD were identified among control newborns during the 1-year follow-up. Group characteristics are summarized in Table 1, and additional clinical data are available in the eAppendix in the Supplement.

**Table 1.** Summary of Patient Characteristics Included in the Study

Characteristic	Mean (SD)		
	Control newborns (n = 81)	Newborns with CHD (n = 34)	Total (N = 115)
Male sex, No. (%)	40 (49)	23 (68)	63 (55)
Cesarean delivery, No. (%)	7 (9)	7 (21)	14 (12)
Gestational age, wk	39.7 (1.4)	39.2 (1.4)	39.6 (1.4)
Birth weight, g	3505 (476)	3391 (555)	3471 (501)
Body surface area, m^2^	0.22 (0.02)	0.22 (0.02)	0.22 (0.02)
Apgar score^a^			
At 1 min	8.8 (0.9)	9.0 (1.2)	8.9 (1.0)
At 5 min	9.9 (0.3)	9.6 (0.9)	9.8 (0.6)
At 10 min	10.0 (0.2)	9.8 (0.5)	9.9 (0.3)

Abbreviation: CHD, congenital heart disease.

^a^ Apgar score ranges from 1 to 10, with a score of 7 or higher considered within normal limits of health for a newborn directly after birth.

### CHD Background in the Study Setting

During the study (July 2018 to June 2020), 2030 to 2041 annual deliveries occurred at the participating hospital, representing approximately 50% of all births in the designated Swedish region (approximately 350 000 inhabitants), and 102 newborns were diagnosed with CHD in the region. Based on annual reports of the Swedish Registry of Congenital Heart Disease, no substantial national changes in CHD incidences have been observed, and 30-day mortality after pediatric cardiac surgery has remained at approximately 2%.^4^ Detection of prenatal CHD has varied in the study region, with greater than 80% of single ventricles identified, whereas detection of coarctation of the aorta has remained challenging with less than 20% of cases identified. Between 2014 and 2017, the combined rate of prenatal CHD diagnoses was 55% in the region (n = 18 of 33 newborns); these diagnoses included single ventricles; atrioventricular septal defects; lesions with large ventricular septal defect and overriding aorta, such as Fallot tetralogy; transposition of the great arteries; severe aortic valve disease; heterotaxy lesions; and Ebstein anomaly. In 8 of 34 newborns (24%), diagnosis of CHD affected delivery planning.

Universal POX screening was implemented in the Jönköping region in 2008, and initial program review of the first 5.5 years of implementation identified 8 newborns with critical cases of CHD. False-negative results in patients with duct-dependent pulmonary circulations were not found. One newborn with duct-dependent systemic circulation was missed. Two critical CHD deaths occurred after POX screening implementation. False-positive POX screening results associated with technical problems or protocol violations (n = 6) were observed primarily in the first year of implementation. The estimated number of echocardiographies needed to identify critical CHD was 6. This number decreased to approximately 3 when other clinically significant cardiovascular findings, such as persistent pulmonary hypertension, in the newborn were taken into account.

### DBS and EDTA Analyses of NT-proBNP Test

**Figure 2.**
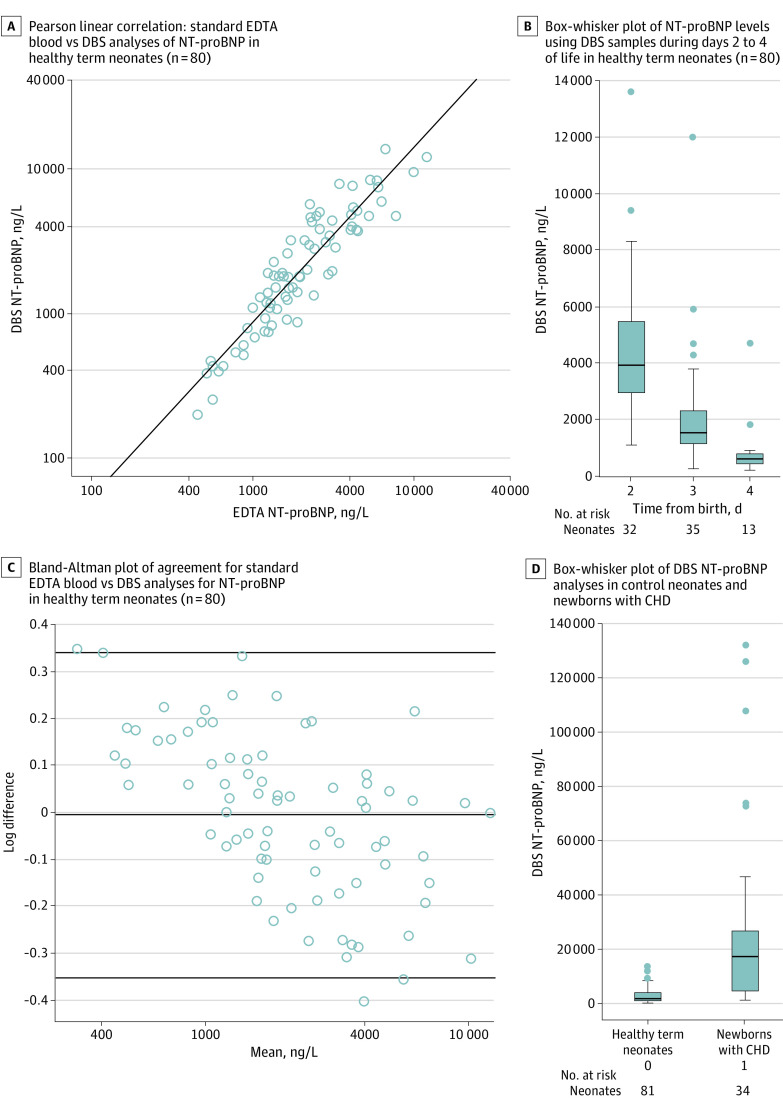
Comparison of Screening Methods for Amino Terminal Fragment of the Prohormone Brain-Type Natriuretic Peptide (NT-proBNP) CHD indicates congenital heart disease; DBS, dried blood spot.

Four of 84 families (5%) either declined to participate in EDTA blood sampling or were deemed by phlebotomy staff as not suitable for blood sampling. A comparison of DBS and EDTA screening performance using Pearson linear correlation analysis showed excellent positive correlation (80 samples; *r* = 0.93; *P* < .01) (Figure 2A). Bland-Altman analysis showed good agreement for standard EDTA blood vs DBS screening for NT-proBNP in healthy term neonates (bias [SD], 3.329 [0.172]; 95% CI, −0.341 to 0.331), making the DBS assay suitable for quantification across a wide range of values (Figure 2C). Median (IQR) DBS values in control newborns decreased in the first few days of life (day 2: 3921 [2946-5475] ng/L; day 3: 1524 [1150-2300] ng/L; day 4: 608 [438-802] ng/L) (Figure 2B). The median (IQR) DBS value was 1900 (1100-4000) ng/L for control newborns and 17 240 (4735-26 940) ng/L for newborns with CHD (Figure 2D). The DBS samples from newborns with CHD showed statistically significantly elevated NT-proBNP levels (*P* < .05). Measured NT-proBNP concentrations in different CHD types are detailed in the eAppendix in the Supplement.

The NT-proBNP cutoff value of greater than 12 000 ng/L in control newborns identified 3 asymptomatic neonates, and cardiac review revealed 1 case of ventricular septal defect and 2 cases of patent ductus arteriosus (PDA) in these neonates that required further cardiac follow-up. Echocardiographic findings were confirmed by a second experienced pediatric cardiologist (P.L.) who was not aware of the initial CHD diagnoses.

Among the 9 initially asymptomatic newborns with CHD after birth who required surgical cardiac treatment within 6 months, 6 (67%) had NT-proBNP levels greater than 12 000 ng/L. Among the 11 asymptomatic newborns with CHD and NT-proBNP levels less than or equal to 12 000 ng/L, 2 (18%) had potentially critical CHD. These newborns had late presentations of coarctation of the aorta and survived to cardiac treatment.

### Combined NT-proBNP Test and POX Screening

**Figure 3.**
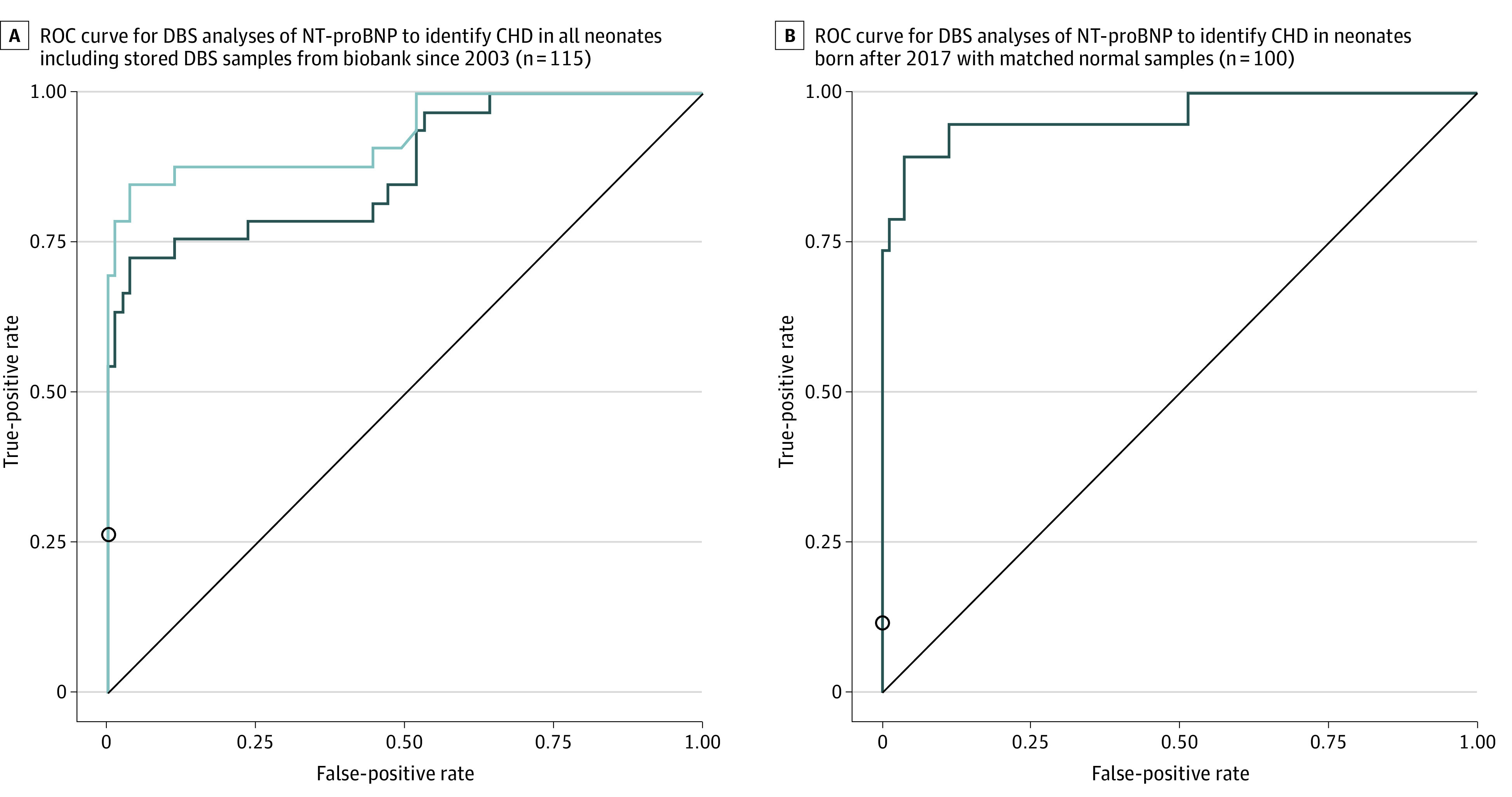
Receiver Operating Characteristic Curves for Dried Blood Spot Analyses of Amino Terminal Fragment of the Prohormone Brain-Type Natriuretic Peptide (NT-proBNP) A, Grey curve indicates NT-proBNP test alone, and the black curve indicates combined NT-pro-BNP test and abnormal pulse oximetry (POX) screening. B, Addition of POX screening (black circle) did not improve the overall screening performance. CHD indicates congenital heart disease.

In this diagnostic study, 24 of 34 CHD cases (71%) and 13 of 19 critical CHD cases (68%) could be identified by elevated DBS NT-proBNP test results alone. When POX screening and NT-proBNP test results were combined, detection of any CHD type improved to 82% (28 of 34 cases) and detection of critical CHD improved to 89% (17 of 19 cases) (Table 2). The DBS assay performed well, achieving an area under the curve (AUC) of the ROC curve of 0.86 (Figure 3A). For the DBS NT-proBNP test alone, the AUC was 0.87 (SE, 0.041; 95% CI, 0.792-0.952; asymptotic *P* < .05). For the combined NT-proBNP test and abnormal POX screening, the AUC was 0.93 (SE, 0.032; 95% CI, 0.865-0.989; asymptotic *P* < .05). The AUC was 0.83 for sampling day 2 (n = 50), 0.92 for sampling day 3 (n = 44), and 0.96 for sampling day 4 (n = 18) (ROC plots not shown in Figure 3). The AUC improved to 0.96 (SE, 0.027; 95% CI, 0.908-1.0; asymptotic *P* < .05) when control newborns were matched to newborns with CHD who were born between July 1, 2018, and May 31, 2019 (Figure 3B). The final ROC plot suggested an optimized NT-proBNP test cutoff for the detection of any CHD at 8550 ng/L (eAppendix in the Supplement).

**Table 2.** Identification of Congenital Heart Disease Cases

Screening method	No. (%)	
	All CHD (n = 34)	Critical CHD (n = 19)
Prenatal diagnosis alone	8 (24)	7 (37)
Postnatal examination alone	18 (53)	12 (63)
Postnatal POX alone	9 (26)	7 (37)
Combined prenatal diagnosis, postnatal examination, and POX	20 (59)	14 (74)
DBS NT-proBNP >8550 ng/L alone	24 (71)	13 (68)
Combined DBS NT-proBNP >8550 ng/L and POX^a^	28 (82)	17 (89)
Cases missed by prenatal and postnatal screening but found by DBS NT-proBNP >8550 ng/L alone	8 of 14 (57)	2 of 5 (40)

Abbreviations: CHD, congenital heart disease; DBS, dried blood spot; NT-proBNP, amino terminal fragment of the prohormone brain-type natriuretic peptide; POX, pulse oximetry.

^a^ Postnatal clinical examination did not result in identification of additional cases.

## Discussion

To our knowledge, this study provides the largest prospective data set on NT-proBNP levels in clinically healthy, term newborns on days 2 to 4 of life; however, the observed variation in control newborns and newborns with CHD needs further exploration to optimize timing and improve cutoff values of DBS NT-proBNP screening. Dried blood spot screening poses several technical challenges owing to hematocrit and conformational protein changes during the drying process.^45,46^ Slow degradation of cardiovascular biomarkers in stored DBS samples over longer periods has been reported.^47^ We assumed a –1% decrease in NT-proBNP levels per year and applied the same DBS sample storage conditions to all the study groups. Still, this assumption may have underestimated the degradation in older samples given that matched batch analyses in DBS samples from 2018 to 2019 led to excellent discrimination compared with older CHD samples.

Although data on gestational age, birth weight, and Apgar scores were similar between groups, male predominance was observed in the CHD group, which is consistent with findings in published reports.^1^ We saw a higher percentage of newborns with CHD who had cesarean delivery, but this study was not designed to evaluate reasons for this finding, and mode of delivery had no implications for test results. Noncardiac disease, such as perinatal hypoxemia, persistent pulmonary hypertension, and sepsis, may be associated with cardiovascular biomarkers.^48,49,50^ Gestational age plays a role in NT-proBNP levels, as demonstrated in premature neonates with and without PDA (the pattern of NT-proBNP levels is higher in neonates with PDA than in those in whom the PDA is closed), and NT-proBNP levels seem to follow a similar postnatal pattern in term neonates (in whom the levels decline over the first few days of life).^51,52^ We did not recruit premature neonates or those who required neonatal inpatient care, populations in which the new test warrants further evaluation.

The need to adhere to guidelines to optimize newborn screening programs has been reviewed.^46^ Maternity staff at the participating hospital largely adhered to the guidelines, and we observed only 1 control newborn whose screening was missed initially and 1 newborn with CHD whose screening was performed more than 3 weeks after birth; neither case altered the overall findings. Because of maternally reported health care evaluations and the costs of inpatient care, a pattern toward early discharge after uncomplicated deliveries has emerged, which may increase the likelihood that CHD goes undetected early after birth because of variations in ductal closure.^53^ Screening programs must adapt to this possibility, and the DBS test may serve as a solution even though NT-proBNP levels remain variable during the initial postnatal phase. The observed downward pattern of NT-proBNP levels in control newborns supports previously published data.^38,54^

The new DBS NT-proBNP test was comparable to current screening standards and minimized blood requirements to 3 μl. We identified 68% of newborns with critical CHD based on elevated NT-proBNP levels alone. Combined POX screening and NT-proBNP test identified 82% of all CHD cases and 89% of critical CHD cases, outperforming current screening methods. These results suggest that cardiovascular biomarkers in neonates can be used for timely and accurate identification of CHD and may even prove valuable in settings with limited health care resources by offering centralized screening for CHD through established newborn screening programs.

### Limitations

This diagnostic study was relatively small, and the timing of DBS screening varied slightly because we wanted to reflect common clinical practice during the first week of life and minimize the need for additional painful blood sampling in newborns. We cannot fully exclude the possibility of selection bias when families were approached for study inclusion. We tried to mitigate this bias by preselecting the days for recruitment without prior knowledge of which control newborns or newborns with CHD were available for enrollment. Echocardiography was not performed in all control newborns because of resource limitations, and we cannot rule out the possibility of minor CHD going unnoticed in this group, but no late CHD presentations were seen in the first year of life.

## Conclusions

In this diagnostic study, the performance of a newly developed DBS screening test for measuring NT-proBNP levels using a minimal amount of blood was examined. Additional reference values were established for days 2 to 4 of life, reflecting the timing of common newborn screening programs. The DBS NT-proBNP test discriminated well between healthy newborns and newborns with various types of CHD, including critical lesions. This new test warrants further investigation in larger neonatal cohorts to evaluate its ability to detect CHD.

## Notes

Supplement. **eAppendix.** Data Collection Process and Eligibility Criteria

## References

[1] GBD 2017 Congenital Heart Disease Collaborators Global, regional, and national burden of congenital heart disease, 1990-2017: a systematic analysis for the Global Burden of Disease Study 2017. Lancet Child Adolesc Health. 2020;4(3):185-200. doi:10.1016/S2352-4642(19)30402-X 31978374 PMC7645774

[2] Hoffman JI The global burden of congenital heart disease. Cardiovasc J Afr. 2013;24(4):141-145. doi:10.5830/CVJA-2013-028 24217047 PMC3721933

[3] Kassebaum N, Kyu HH, Zoeckler L, ; Global Burden of Disease Child and Adolescent Health Collaboration Child and adolescent health from 1990 to 2015: findings from the Global Burden of Diseases, Injuries, and Risk Factors 2015 Study. JAMA Pediatr. 2017;171(6):573-592. doi:10.1001/jamapediatrics.2017.0250 28384795 PMC5540012

[4] Lundström NR, Berggren H, Björkhem G, Jögi P, Sunnegârdh J Centralization of pediatric heart surgery in Sweden. Pediatr Cardiol. 2000;21(4):353-357. doi:10.1007/s002460010079 10865012

[5] Chew C, Halliday JL, Riley MM, Penny DJ Population-based study of antenatal detection of congenital heart disease by ultrasound examination. Ultrasound Obstet Gynecol. 2007;29(6):619-624. doi:10.1002/uog.4023 17523161

[6] Chew C, Stone S, Donath SM, Penny DJ Impact of antenatal screening on the presentation of infants with congenital heart disease to a cardiology unit. J Paediatr Child Health. 2006;42(11):704-708. doi:10.1111/j.1440-1754.2006.00955.x 17044898

[7] Cloete E, Bloomfield FH, Sadler L, de Laat MWM, Finucane AK, Gentles TL Antenatal detection of treatable critical congenital heart disease is associated with lower morbidity and mortality. J Pediatr. 2019;204:66-70. doi:10.1016/j.jpeds.2018.08.056 30292491

[8] McBrien A, Sands A, Craig B, Dornan J, Casey F Major congenital heart disease: antenatal detection, patient characteristics and outcomes. J Matern Fetal Neonatal Med. 2009;22(2):101-105. doi:10.1080/14767050802483106 19085626

[9] Peake LK, Draper ES, Budd JL, Field D Outcomes when congenital heart disease is diagnosed antenatally versus postnatally in the UK: a retrospective population-based study. BMC Pediatr. 2015;15:58. doi:10.1186/s12887-015-0370-3 25982522 PMC4470120

[10] Uzun O, Kennedy J, Davies C, Training: improving antenatal detection and outcomes of congenital heart disease. BMJ Open Qual. 2018;7(4):e000276. doi:10.1136/bmjoq-2017-000276 30555930 PMC6267317

[11] Eckersley L, Sadler L, Parry E, Finucane K, Gentles TL Timing of diagnosis affects mortality in critical congenital heart disease. Arch Dis Child. 2016;101(6):516-520. doi:10.1136/archdischild-2014-307691 26130379

[12] Brown KL, Ridout DA, Hoskote A, Verhulst L, Ricci M, Bull C Delayed diagnosis of congenital heart disease worsens preoperative condition and outcome of surgery in neonates. Heart. 2006;92(9):1298-1302. doi:10.1136/hrt.2005.078097 16449514 PMC1861169

[13] Cloete E, Bloomfield FH, Cassells SA, de Laat MWM, Sadler L, Gentles TL Newborn pulse oximetry screening in the context of a high antenatal detection rate of critical congenital heart disease. Acta Paediatr. 2020;109(1):93-99. doi:10.1111/apa.14946 31332832 PMC6972642

[14] Aranguren Bello HC, Londoño Trujillo D, Troncoso Moreno GA, Oximetry and neonatal examination for the detection of critical congenital heart disease: a systematic review and meta-analysis. F1000Res. 2019;8:242. doi:10.12688/f1000research.17989.1 31372214 PMC6659768

[15] Mawson IE, Babu PL, Simpson JM, Fox GF Pulse oximetry findings in newborns with antenatally diagnosed congenital heart disease. Eur J Pediatr. 2018;177(5):683-689. doi:10.1007/s00431-018-3093-2 29404717 PMC5899118

[16] Plana MN, Zamora J, Suresh G, Fernandez-Pineda L, Thangaratinam S, Ewer AK Pulse oximetry screening for critical congenital heart defects. Cochrane Database Syst Rev. 2018;3:CD011912. doi:10.1002/14651858.CD011912.pub2 29494750 PMC6494396

[17] Mahle WT, Newburger JW, Matherne GP, ; American Heart Association Congenital Heart Defects Committee of the Council on Cardiovascular Disease in the Young, Council on Cardiovascular Nursing, and Interdisciplinary Council on Quality of Care and Outcomes Research; American Academy of Pediatrics Section on Cardiology and Cardiac Surgery, and Committee on Fetus and Newborn Role of pulse oximetry in examining newborns for congenital heart disease: a scientific statement from the American Heart Association and American Academy of Pediatrics. Circulation. 2009;120(5):447-458. doi:10.1161/CIRCULATIONAHA.109.192576 19581492

[18] de Wahl Granelli A, Mellander M, Sunnegårdh J, Sandberg K, Ostman-Smith I Screening for duct-dependant congenital heart disease with pulse oximetry: a critical evaluation of strategies to maximize sensitivity. Acta Paediatr. 2005;94(11):1590-1596. doi:10.1111/j.1651-2227.2005.tb01834.x 16381094

[19] Lannering K, Bartos M, Mellander M Late diagnosis of coarctation despite prenatal ultrasound and postnatal pulse oximetry. Pediatrics. 2015;136(2):e406-e412. doi:10.1542/peds.2015-1155 26169432

[20] Jawin V, Ang HL, Omar A, Thong MK Beyond critical congenital heart disease: newborn screening using pulse oximetry for neonatal sepsis and respiratory diseases in a middle-income country. PLoS One. 2015;10(9):e0137580. doi:10.1371/journal.pone.0137580 26360420 PMC4567069

[21] McClain MR, Hokanson JS, Grazel R, Critical congenital heart disease newborn screening implementation: lessons learned. Matern Child Health J. 2017;21(6):1240-1249. doi:10.1007/s10995-017-2273-4 28092064 PMC5663229

[22] Johnson LC, Lieberman E, O’Leary E, Geggel RL Prenatal and newborn screening for critical congenital heart disease: findings from a nursery. Pediatrics. 2014;134(5):916-922. doi:10.1542/peds.2014-1461 25287457

[23] Andersen NJ, Mondal TK, Preissler MT, Detection of immunoglobulin isotypes from dried blood spots. J Immunol Methods. 2014;404:24-32. doi:10.1016/j.jim.2013.12.001 24333851 PMC4663688

[24] Scott CR, Elliott S, Buroker N, Identification of infants at risk for developing Fabry, Pompe, or mucopolysaccharidosis-I from newborn blood spots by tandem mass spectrometry. J Pediatr. 2013;163(2):498-503. doi:10.1016/j.jpeds.2013.01.031 23465405 PMC3725184

[25] Lobitz S, Telfer P, Cela E, ; with the endorsement of EuroBloodNet, the European Reference Network in Rare Haematological Diseases Newborn screening for sickle cell disease in Europe: recommendations from a Pan-European Consensus Conference. Br J Haematol. 2018;183(4):648-660. doi:10.1111/bjh.15600 30334577

[26] Castellani C, Massie J, Sontag M, Southern KW Newborn screening for cystic fibrosis. Lancet Respir Med. 2016;4(8):653-661. doi:10.1016/S2213-2600(16)00053-9 27053341

[27] Pollitt RJ, Green A, McCabe CJ, Neonatal screening for inborn errors of metabolism: cost, yield and outcome. Health Technol Assess. 1997;1(7):i-iv, 1-202. doi:10.3310/hta1070 9483160

[28] Pandor A, Eastham J, Beverley C, Chilcott J, Paisley S Clinical effectiveness and cost-effectiveness of neonatal screening for inborn errors of metabolism using tandem mass spectrometry: a systematic review. Health Technol Assess. 2004;8(12):iii, 1-121. doi:10.3310/hta8120 14982654

[29] Ponikowski P, Voors AA, Anker SD, ; ESC Scientific Document Group 2016 ESC guidelines for the diagnosis and treatment of acute and chronic heart failure: the task force for the diagnosis and treatment of acute and chronic heart failure of the European Society of Cardiology (ESC) developed with the special contribution of the Heart Failure Association (HFA) of the ESC. Eur Heart J. 2016;37(27):2129-2200. doi:10.1093/eurheartj/ehw128 27206819

[30] Cantinotti M, Giordano R, Scalese M, Prognostic role of BNP in children undergoing surgery for congenital heart disease: analysis of prediction models incorporating standard risk factors. Clin Chem Lab Med. 2015;53(11):1839-1846. doi:10.1515/cclm-2014-1084 25901715

[31] Cantinotti M, Storti S, Ripoli A, Diagnostic accuracy of B-type natriuretic hormone for congenital heart disease in the first month of life. Clin Chem Lab Med. 2010;48(9):1333-1338. doi:10.1515/CCLM.2010.251 20560803

[32] Cantinotti M, Walters HL, Crocetti M, Marotta M, Murzi B, Clerico A BNP in children with congenital cardiac disease: is there now sufficient evidence for its routine use? Cardiol Young. 2015;25(3):424-437. doi:10.1017/S1047951114002133 25601330

[33] Davlouros PA, Karatza AA, Xanthopoulou I, Diagnostic role of plasma BNP levels in neonates with signs of congenital heart disease. Int J Cardiol. 2011;147(1):42-46. doi:10.1016/j.ijcard.2009.07.029 19712988

[34] El-Khuffash A, Molloy EJ Are B-type natriuretic peptide (BNP) and N-terminal-pro-BNP useful in neonates? Arch Dis Child Fetal Neonatal Ed. 2007;92(4):F320-F324. doi:10.1136/adc.2006.106039 17585100 PMC2675431

[35] Kulkarni M, Gokulakrishnan G, Price J, Fernandes CJ, Leeflang M, Pammi M Diagnosing significant PDA using natriuretic peptides in preterm neonates: a systematic review. Pediatrics. 2015;135(2):e510-e525. doi:10.1542/peds.2014-1995 25601976

[36] Lowenthal A, Camacho BV, Lowenthal S, Usefulness of B-type natriuretic peptide and N-terminal pro-B-type natriuretic peptide as biomarkers for heart failure in young children with single ventricle congenital heart disease. Am J Cardiol. 2012;109(6):866-872. doi:10.1016/j.amjcard.2011.10.049 22196786 PMC3294194

[37] Moses EJ, Mokhtar SAI, Hamzah A, Abdullah BS, Yusoff NM Usefulness of N-terminal-pro-B-type natriuretic peptide as a screening tool for identifying pediatric patients with congenital heart disease. Laboratory Medicine. 2011;42(2):75-80. doi:10.1309/LMW0U87COTHXGELF

[38] Cantinotti M, Storti S, Parri MS, Prontera C, Murzi B, Clerico A Reference intervals for brain natriuretic peptide in healthy newborns and infants measured with an automated immunoassay platform. Clin Chem Lab Med. 2010;48(5):697-700. doi:10.1515/CCLM.2010.129 20187851

[39] Massimiliano C, Simona S, Bruno M, Aldo C, Das BB Clinical relevance of different B-type natriuretic peptide decisional cutoff values for the diagnosis of congenital heart disease in the first weeks of life. Pediatr Cardiol. 2011;32(4):537. doi:10.1007/s00246-011-9898-7 21298426

[40] Mir TS, Flato M, Falkenberg J, Plasma concentrations of N-terminal brain natriuretic peptide in healthy children, adolescents, and young adults: effect of age and gender. Pediatr Cardiol. 2006;27(1):73-77. doi:10.1007/s00246-005-1022-4 16132298

[41] World Medical Association World Medical Association Declaration of Helsinki: ethical principles for medical research involving human subjects. JAMA. 2013;310(20):2191-2194. doi:10.1001/jama.2013.281053 24141714

[42] SWEDCON Årsrapporter. Accessed October 23, 2020. https://www.ucr.uu.se/swedcon/arsrapporter

[43] Albers S, Mir TS, Haddad M, Läer S N-Terminal pro-brain natriuretic peptide: normal ranges in the pediatric population including method comparison and interlaboratory variability. Clin Chem Lab Med. 2006;44(1):80-85. doi:10.1515/CCLM.2006.016 16375591

[44] Thilén U, Björkhem G, Jeremiasen I, Synnergren M, Bergman G Annual Report of the Swedish National Patient Register on Congenital Heart Disease. SWEDCON; 2018:44-45.

[45] Eshghi A, Pistawka AJ, Liu J, Concentration determination of >200 proteins in dried blood spots for biomarker discovery and validation. Mol Cell Proteomics. 2020;19(3):540-553. doi:10.1074/mcp.TIR119.001820 31896676 PMC7050112

[46] Lim MD Dried blood spots for global health diagnostics and surveillance: opportunities and challenges. Am J Trop Med Hyg. 2018;99(2):256-265. doi:10.4269/ajtmh.17-0889 29968557 PMC6090344

[47] Björkesten J, Enroth S, Shen Q, Stability of proteins in dried blood spot biobanks. Mol Cell Proteomics. 2017;16(7):1286-1296. doi:10.1074/mcp.RA117.000015 28501802 PMC5500761

[48] Heindel K, Holdenrieder S, Patel N, Early postnatal changes of circulating N-terminal-pro-B-type natriuretic peptide in neonates with congenital diaphragmatic hernia. Early Hum Dev. 2020;146:105049. doi:10.1016/j.earlhumdev.2020.105049 32402829

[49] Cetin I, Kantar A, Unal S, Cakar N The assessment of time-dependent myocardial changes in infants with perinatal hypoxia. J Matern Fetal Neonatal Med. 2012;25(9):1564-1568. doi:10.3109/14767058.2011.644365 22122298

[50] Vijlbrief DC, Benders MJ, Kemperman H, van Bel F, de Vries WB Cardiac biomarkers as indicators of hemodynamic adaptation during postasphyxial hypothermia treatment. Neonatology. 2012;102(4):243-248. doi:10.1159/000339117 22907615

[51] Tauber KA, Doyle R, Granina E, Munshi U B-type natriuretic peptide levels normalise in preterm infants without a patent ductus arteriosus by the fifth postnatal day. Acta Paediatr. 2016;105(8):e352-e355. doi:10.1111/apa.13480 27206680

[52] Buddhe S, Dhuper S, Kim R, NT-proBNP levels improve the ability of predicting a hemodynamically significant patent ductus arteriosus in very low-birth-weight infants. J Clin Neonatol. 2012;1(2):82-86. doi:10.4103/2249-4847.96758 24027696 PMC3743145

[53] Johansson M, Thies-Lagergren L, Wells MB Mothers’ experiences in relation to a new Swedish postnatal home-based model of midwifery care-a cross-sectional study. Midwifery. 2019;78:140-149. doi:10.1016/j.midw.2019.07.010 31446229

[54] Cantinotti M, Passino C, Storti S, Ripoli A, Zyw L, Clerico A Clinical relevance of time course of BNP levels in neonates with congenital heart diseases. Clin Chim Acta. 2011;412(23-24):2300-2304. doi:10.1016/j.cca.2011.08.030 21910979

