# Supplementary material for: Evaluation of Circulating Cardiovascular Biomarker Levels for Early Detection of Congenital Heart Disease in Newborns in Sweden
Source: JAMA Netw Open. 2020 Dec 2;3(12):e2027561. doi: 10.1001/jamanetworkopen.2020.27561 (PMC7711323; doi:10.1001/jamanetworkopen.2020.27561)
Supplement: Supplement. — eAppendix. Data Collection Process and Eligibility Criteria [file jamanetwopen-e2027561-s001.pdf]

Supplemental Online Content

Clausen H, Norén E, Valtonen S, Koivu A, Sairanen M, Liuba P. Evaluation of circulating cardiovascular biomarker levels for early detection of congenital heart disease in newborns in Sweden. *JAMA Netw Open*. 2020;3(12):e2027561. doi:10.1001/jamanetworkopen.2020.27561

eAppendix. Data Collection Process and Eligibility Criteria

This supplemental material has been provided by the authors to give readers additional information about their work.

© 2020 Clausen H et al. *JAMA Network Open.*

eAppendix. Data Collection Process and Eligibility Criteria

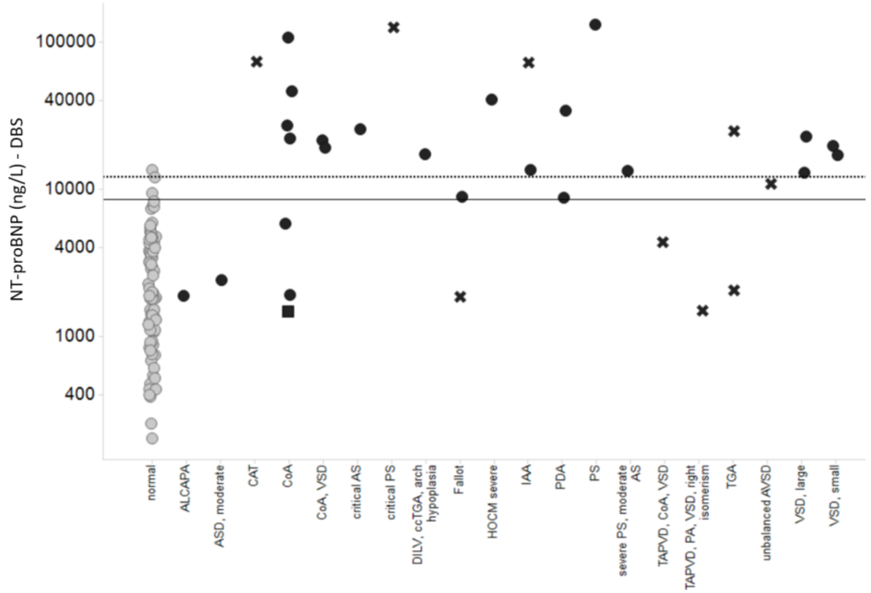

<u>Controls & CHD cases:</u> NT-pro-BNP levels (ng/l) in various types of CHD versus normal controls. Dashed line = initial cut-off at 12,000 ng/l. solid line = refined cut-off at 8,550 ng/l based on ROC analyses.
CHD cases (black) and normal controls (grey) included in DBS analyses of NT-pro-BNP (n=115): Cross = failed POX screening (cyanosis) in CHD cases; square = no POX (born prior to screening). Initial cut-off value for NT-pro-BNP (dotted line) of 12,000 ng/l and revised cut-off derived from RCO at 8,550 ng/l are shown.
ALCAPA = anomalous left coronary artery from the pulmonary artery, AS = aortic stenosis, ASD = atrial septal defect, AVSD = atrioventricular septal defect, CAT = common arterial trunk, ccTGA = congenitally corrected TGA, CoA = coarctation of the aorta, DILV = double inlet left ventricle, HOCM = hypertrophic obstructive cardiomyopathy, IAA = interrupted aortic arch, PA = pulmonary atresia, PDA = persistent ductus arteriosus, PS = pulmonary stenosis, TAPVD = total anomalous pulmonary venous drainage, TGA = transposition of the great arteries, VSD = ventricular septal defect.

© 2020 Clausen H et al. JAMA Network Open.

Data collection criteria:

Study ID
NT-pro-BNP (EDTA blood) in ng/l
NT-pro-BNP (DBS) in ng/l
Date & time of blood sampling (EDTA & DBS in controls)
Date & time of blood sampling (DBS in CHD cases)
Age at sampling (days of life)
Type of delivery (vaginal, section)
Date & time of birth
Gender (male/female)
Birth weight (kg) & length (cm)
Gestational age (weeks)
Apgar scores at 1, 5, 10 minutes
POX screening result (normal / abnormal)
‘First day baby check’ clinical examination of cardiovascular system (normal / abnormal)
Any cardiac symptoms within 1 month, 6 months, 12 months of delivery
Name of cardiac condition (based on WHO ICD-10 diagnoses codes)
Open heart surgery or cardiac catheter intervention (accepted for or performed during follow-up)
Prenatally suspected cardiac lesion with impact on delivery planning

Eligibility criteria:

Exclusion criteria for controls

Inability to obtain written informed consent
Prematurity
Additional postnatal neonatal in-patient care requirements (non-maternity ward care)
Syndromic, dysmorphic or other medical healthcare concerns necessitating paediatric follow-up
Enrolment in other research study that may present ethical or other practical conflicts
No intension to participate in blood sampling during the first week of life as per national neonatal screening program
No access to electronic patient records for data acquisition and quality control

© 2020 Clausen H et al. JAMA Network Open.

Exclusion criteria for CHD cases

Inability to obtain written informed consent
Prematurity
Patient age over 17 years at time of enrolment
Enrolment in other research study that may present ethical or other practical conflicts
No previous participation in Swedish national neonatal screening program and no stored dried blood sample available through national screening laboratory
No routinely scheduled paediatric cardiology out-patient clinic review during study period in studied region to discuss study participation / enrolment
No access to electronic patient records for data acquisition and quality control

**heal thy**	**NT- pro-**	**NT- pro**	**Age at**	**Type of**	**ge nd**	**bir th**	**bir th**	**gesta tiona**	**AP GA**	**AP GA**	**AP GA**	**norm al**	**normal cardiov**	**cardiac sympt**	**cardiac sympt**	**heart surger**	**cut -off**	**prenatally suspected CHD with impact on place of delivery YES=1**

© 2020 Clausen H et al. JAMA Network Open.

= 0 diseased with CHD = 1	BNP (ng/L, EDTA blood standard)	-BNP (ng/L, DBS)	sampling (days of life)	delivery 0=vaginal 1=sectio 2=unknown	er (1=M, 0=F)	weight (gram)	length (cm)	l age in weeks	R at 1 min	R at 5 min	R at 10 min	POX screening YES=1 NO=0 UNKNOWN (born prior to universal screening) =2	ascular 'baby check' screening YES=1 No=0 UNKNOWN=2	oms within 1 month NO=0 YES=1 UNKNOWN=2	oms within 6 months NO=0 YES=1 UNKNOWN=2	y (accepted for or undergone) YES=1 NO=0 UNKNOWN=2 catheter intervention (accepted for or undergone) =3	for clinical call back and acute review with echo >12000 ng/l: YES=1, NO=0, known CHD case=2	NO=0
0	5959	7411	2	0	1	2730	47	37	10	10	10	1	1	0	0	0	0	0
0	5812	824	2	0	1	43	54	39	10	10	10	1	1	0	0	0	0	0

© 2020 Clausen H et al. JAMA Network Open.

		2				20												
0	1441	1811	3	0	0	3710	51	39	10	10	10	1	1	0	0	0	0	0
0	1371	1524	3	0	1	3210	49	40	10	10	10	1	1	0	0	0	0	0
0	2573	3843	2	1	1	3680	50	39	9	10	10	1	1	0	0	0	0	0
0	3079	1971	3	0	1	3105	49	41	9	10	10	1	1	0	0	0	0	0
0	7696	4694	3	0	0	3985	52	41	9	10	10	1	1	0	0	0	0	0
0	1584	1316	3	0	0	3490	49	41	9	10	10	1	1	0	0	0	0	0
0	1402	1075	3	0	0	3770	51	41	5	10	10	1	1	0	0	0	0	0
0	1654	1509	3	0	0	3240	48	40	9	10	10	1	1	0	0	0	0	0
0	2220	2974	2	1	1	3500	50	39	9	10	10	1	1	0	0	0	0	0
0	1622	1252	3	0	1	3435	53	40	9	10	10	1	1	0	0	0	0	0
0	1226	1900	3	0	1	3910	52	39	9	10	10	1	1	0	0	0	0	0
0	514	389	4	0	1	3460	52	40	9	10	10	1	1	0	0	0	0	0
0	774	545	4	0	0	2680	48	39	9	10	10	1	1	0	0	0	0	0
0	1877	872	3	0	0	3630	50	39	9	10	10	1	1	0	0	0	0	0
0	2992	3250	2	0	1	4000	53	41	9	10	10	1	1	0	0	0	0	0
0	1240	750	3	0	1	3970	51	40	9	10	10	1	1	0	0	0	0	0

© 2020 Clausen H et al. JAMA Network Open.

0	1866	1413	3	0	1	4270	51	40	8	10	10	1	1	0	0	0	0	0
0	1918	1813	3	0	1	4115	51	42	9	9	10	1	1	0	0	0	0	0
0	1021	688	4	0	0	4200	50	39	8	10	10	1	1	0	0	0	0	0
0	1296	834	3	0	0	2960	48	40	9	10	10	1	1	0	0	0	0	0
0	560	256	3	0	0	3345	50	42	9	10	10	1	1	0	0	0	0	0
0	1759	1514	3	0	1	3270	52	38	8	9	10	1	1	0	0	0	0	0
0	543	475	4	0	0	3450	50	40	9	10	10	1	1	0	0	0	0	0
0	2902	1876	2	0	1	3860	51	42	8	10	10	1	1	0	0	0	0	0
0	1179	932	3	0	0	3500	51	41	9	10	10	1	1	0	0	0	0	0
0	450	202	4	0	0	2640	49	39	9	10	10	1	1	0	0	0	0	0
0	1614	909	4	0	1	2865	49	37	9	10	10	1	1	0	0	0	0	0
0	4003	3797	2	1	0	2790	48	38	8	10	10	1	1	0	0	0	0	0
0	4443	3696	2	1	1	3480	50	38	9	10	10	1	1	0	0	0	0	0
0	1176	757	4	0	1	3260	49	40	9	10	10	1	1	0	0	0	0	0
0	3087	4371	2	0	0	3370	49	41	9	10	10	1	1	0	0	0	0	0
0	3222	2860	2	0	1	3260	50	40	10	10	10	1	1	0	0	0	0	0
0	651	436	3	0	0	36	50	40	9	10	10	1	1	0	0	0	0	0

© 2020 Clausen H et al. JAMA Network Open.

						50												
0	1271	1189	2	0	1	4125	55	41	9	10	10	1	1	0	0	0	0	0
0	2367	1340	3	0	0	4270	53	42	9	10	10	1	1	0	0	0	0	0
0	868	608	4	0	0	3090	48	38	9	9	9	1	1	0	0	0	0	0
0	556	438	4	0	0	3255	51	39	9	9	10	1	1	0	0	0	0	0
0	2954	3442	2	0	1	3510	53	41	9	10	10	1	1	0	0	0	0	0
0	918	802	4	0	0	3130	48	38	9	10	10	1	1	0	0	0	0	0
0	1337	2286	2	0	1	3780	54	40	9	10	9	1	1	0	0	0	0	0
0	1537	1815	3	0	0	2450	46	37	10	10	10	1	1	0	0	0	0	0
0	1333	1840	3	0	0	3725	52	39	9	10	10	1	1	0	0	0	0	0
0	no sample	3437	3	0	1	3480	52	39	9	10	10	1	1	0	0	0	0	0
0	no sample	2115	3	0	0	3700	52	41	9	10	10	1	1	0	0	0	0	0
0	869	519	3	0	0	3390	50	40	9	10	10	1	1	0	0	0	0	0
0	2079	3209	2	1	0	2800	48	40	9	10	10	1	1	0	0	0	0	0
0	no sample	3577	2	0	0	2600	50	37	9	10	10	1	1	0	0	0	0	0

© 2020 Clausen H et al. JAMA Network Open.

0	6621	13600	2	0	1	3640	52	40	9	10	10	1	1	0	0	0	0	0
0	9829	9400	2	1	1	3670	49	41	9	10	10	1	1	0	0	0	0	0
0	4367	3800	3	0	0	3340	50	39	9	9	9	1	1	0	0	0	0	0
0	2293	4300	3	0	1	3510	49	41	8	10	10	1	1	0	0	0	0	0
0	1637	1800	2	0	0	3730	52	39	10	10	10	1	1	0	0	0	0	0
0	4084	4000	2	0	0	3680	50	39	9	10	10	1	1	0	0	0	0	0
0	4422	5100	2	0	1	3260	50	39	9	10	10	1	1	0	0	0	0	0
0	1480	1800	4	0	0	2835	48	40	8	10	10	1	1	0	0	0	0	0
0	1504	1900	3	0	0	2590	48	39	8	10	10	1	1	0	0	0	0	0
0	6240	5900	3	1	1	3520	49	40	9	9	10	1	1	0	0	0	0	0
0	2383	2800	2	0	0	4230	51	41	8	10	10	1	1	0	0	0	0	0
0	1951	1800	4	0	1	4055	53	41	6	10	10	1	1	0	0	0	0	0
0	1698	3200	3	0	1	4035	52	40	9	10	10	1	1	0	0	0	0	0
0	1222	1400	2	0	0	3365	51	40	10	10	10	1	1	0	0	0	0	0
0	1622	2600	3	0	0	3530	51	40	9	10	10	1	1	0	0	0	0	0
0	604	400	4	0	1	4420	54	42	9	10	10	1	1	0	0	0	0	0
0	4176	540	2	0	1	34	49	39	9	9	10	1	1	0	0	0	0	0

© 2020 Clausen H et al. JAMA Network Open.

		0				20												
0	11949	12000	3	0	1	3845	53	41	9	10	10	1	1	0	0	0	0	0
0	4046	4800	2	0	1	4220	52	41	9	10	10	1	1	0	0	0	0	0
0	1263	1100	2	0	0	3390	48	39	6	10	10	1	1	0	0	0	0	0
0	985	1100	3	0	1	4900	55	42	9	10	10	1	1	0	0	0	0	0
0	2815	3100	3	0	0	3670	51	40	9	10	10	1	1	0	0	0	0	0
0	2575	5000	2	0	0	3380	50	41	9	10	10	1	1	0	0	0	0	0
0	3425	7800	2	0	1	2770	49	38	6	10	10	1	1	0	0	0	0	0
0	4137	7600	2	0	1	2780	49	38	9	10	10	1	1	0	0	0	0	0
0	5306	8300	1	0	1	3510	49	41	9	10	10	1	1	0	0	0	0	0
0	2160	2000	3	0	1	3350	50	42	9	10	10	1	1	0	0	0	0	0
0	1099	1300	3	0	0	3795	50	40	9	10	10	1	1	0	0	0	0	0
0	2255	4600	2	0	0	3660	52	39	9	10	10	1	1	0	0	0	0	0
0	1200	1200	3	0	1	3545	52	40	8	10	10	1	1	0	0	0	0	0
0	2448	4700	2	0	0	3125	51	37	7	10	10	1	1	0	0	0	0	0
0	2248	5700	2	0	0	3450	50	38	9	10	10	1	1	0	0	0	0	0
1	12150	8800	3	0	0	3120	48	40	10	10	10	1	1	0	0	0	1	0

© 2020 Clausen H et al. JAMA Network Open.

1	25797	34400	2	0	0	3320	49	40	9	10	10	1	1	0	0	0	1	0
1	16288	19900	3	0	1	4200	55	41	9	10	10	1	1	0	0	0	1	0
1	no sample	125869	3	0	1	3000	47	39	8	9	9	0	0	1	0	1	2	0
1	no sample	46800	2	0	1	2835	46	39	9	10	10	1	0	1	0	1	2	0
1	no sample	24931	3	0	1	3090	50	39	9	10	10	0	0	1	0	1	2	0
1	no sample	8961	2	0	1	3750	50	40	9	10	10	1	0	1	0	1	2	0
1	no sample	5830	4	0	1	3080	48	39	10	10	10	1	1	0	1	1	2	0
1	no sample	25652	2	0	1	2930	47	39	10	10	10	1	0	1	0	1	2	0
1	no sample	107829	3	1	1	3085	48	37	10	10	10	1	0	1	0	1	2	0
1	no sample	27369	2	0	1	4440	55	41	9	10	10	1	1	0	0	1	2	0
1	no sample	22812	4	0	0	3030	49	40	10	10	10	1	1	1	0	1	2	0
1	no sam	72611	3	1	1	2505	50	39	9	10	10	0	1	1	0	1	2	0

© 2020 Clausen H et al. JAMA Network Open.

	ple																	
1	no sample	73443	2	1	1	3710	52	40	8	9	10	0	0	0	0	1	2	1
1	no sample	1889	1	0	1	2960	48	39	10	10	10	1	1	1	0	0	2	0
1	no sample	2065	20	0	0	3320	50	40	9	9	9	0	0	0	0	1	2	1
1	no sample	40587	2	0	0	4360	52	38	9	10	10	1	0	1	0	0	2	0
1	no sample	132120	3	0	1	3150	47	37	8	10	10	1	0	1	0	3	2	0
1	no sample	1480	4	0	1	3820	49	38	9	10	10	2	1	0	0	3	2	0
1	no sample	10960	5	1	1	3880	53	39	9	10	10	0	0	0	0	1	2	1
1	no sample	17180	3	1	1	2416	47	38	3	5	8	1	0	1	0	0	2	0
1	no sample	2410	3	1	0	2405	46	36	10	10	10	1	1	0	0	3	2	0
1	no sample	21440	3	0	1	3750	51	38	9	9	10	1	1	0	1	1	2	0
1	no sam	13380	3	0	0	3460	49	40	9	9	10	1	1	1	0	3	2	0

© 2020 Clausen H et al. JAMA Network Open.

	ple																	
1	no sample	1900	4	0	1	4220	52	41	9	10	10	1	1	0	0	3	2	0
1	no sample	22180	3	0	0	3835	50	38	9	10	10	1	1	0	0	0	2	1
1	no sample	4370	3	0	1	3460	50	41	9	9	9	0	0	0	0	1	2	0
1	no sample	13470	3	0	1	4160	54	40	9	10	10	1	0	0	0	1	2	1
1	no sample	19300	2	0	1	3344	50	39	9	10	10	1	0	0	0	1	2	1
1	no sample	1850	3	1	1	3760	50	39	9	10	10	0	0	0	0	1	2	0
1	no sample	1880	3	0	1	3180	53	40	9	10	10	1	1	0	0	1	2	0
1	no sample	12940	4	0	0	2880	48	41	10	10	10	1	1	0	0	1	2	0
1	no sample	17300	2	0	0	3990	50	41	9	10	10	1	0	0	0	1	2	1
1	no sample	1500	3	0	0	2850	48	38	9	9	9	0	0	0	0	1	2	1

© 2020 Clausen H et al. JAMA Network Open.
